# Unmasking diphtheria in Nigeria: A multifaceted approach to tackle outbreaks and improve immunization rates among the Nigerian population—An updated correspondence

**DOI:** 10.1002/hsr2.1804

**Published:** 2024-01-09

**Authors:** Malik O. Oduoye, Mohammed Dheyaa Marsool Marsool, Muhammad Usman Haider, Dattatreya Mukherjee, Aimen Waqar Khan, Temidayo O. Faloye, Minal Jehan, Abdullahi A. Adegoke, Karim A. Karim, Tirth Dave

**Affiliations:** ^1^ Department of Research Medical Research Circle (MedReC) Bukavu DR Congo; ^2^ Al‐Kindy College of Medicine University of Baghdad Baghdad Iraq; ^3^ Mayo Hospital Lahore King Edward Medical University Lahore Lahore Pakistan; ^4^ Department of Community Medicine Raiganj Government Medical College and Hospital Raiganj India; ^5^ Department of Medicine Jinnah Sindh Medical University Karachi Pakistan; ^6^ Department of Microbiology Federal University of Technology Akure Nigeria; ^7^ Karachi Medical and Dental College Karachi Pakistan; ^8^ Department of Pharmacognosy University of Ibadan Ibadan Nigeria; ^9^ Kamuzu University of Health Sciences Blantyre Malawi; ^10^ Bukovinian State Medical University Chernivtsi Ukraine

**Keywords:** challenges, diphtheria, efforts, Nigeria, prevalence, vaccination

## Abstract

**Background and aims:**

The World Health Organization has recently declared the frequent outbreaks of diphtheria in Nigeria as a public health concern. Although vaccination efforts have been successful in Nigeria, unfortunately, the recent 2023 outbreak in Nigeria has been nothing short of distressing. Of course, cases of diphtheria incidence are under‐reported in Nigeria. This present article aims to proffer a possible multifaceted approach to tackle outbreaks of diphtheria in Nigeria and improve immunization rates against the disease among the Nigerian population

**Methods:**

In writing this study, literature search was done about diphtheria in Nigeria using the following keywords: “diphtheria, prevalence, vaccination, efforts, challenges, and Nigeria” on PubMed, Web of Science, Google Scholar, and ResearchGate within 10 years.

**Result:**

This study found that an estimated seven million people remain unvaccinated and are at risk for infection in the country, especially people living in the Northern part of the country. Between the June 30 and August 31, 2023, Nigeria recorded an unusual increase in the number of confirmed cases of diphtheria, where a total of 5898 suspected cases were reported from 59 local government areas in 11 states across Nigeria. The majority (99.4%) of suspected cases of the disease were reported from six states: Kano (1816), Katsina (234), Yobe (158), Bauchi (79), Kaduna (45), and Borno (33).

**Conclusion:**

If Nigeria is to emerge beyond these frequent epidemics of diphtheria, the Nigerian government must work on tackling this issue on multiple fronts simultaneously, that is, at the national and international levels, as we believe that these levels would give a holistic way to unmask diphtheria in Nigeria.

## 1 INTRODUCTION

Diphtheria is a potentially fatal infectious disease caused by the toxigenic *Corynebacterium* species. It is transmitted via respiratory droplets and by direct contact with infected lesions. As a result, a spectrum of findings ranging from cutaneous to respiratory manifestations going so far as to cause neurologic and cardiac complications exist.^1^ Diphtheria is two types, respiratory diphtheria and cutaneous diphtheria. Respiratory diphtheria can be divided in to nasal diphtheria, pharyngeal diphtheria, and laryngeal diphtheria. In severe case, diphtheria antitoxin (DAT) can be given as a treatment. Although diphtheria vaccinations have significantly reduced morbidity and mortality worldwide, low‐middle‐income countries for example, Nigeria, continue to frequently face outbreaks with a case fatality rate (CFR) reaching up to 40% in certain instances.^2^ For these nations, a much more serious plague persists–the plague of misinformation. Public fallacies like the persistence of immunity from the initial vaccine without the need for booster doses, the acquisition of immunity against colonization, and lack of awareness towards asymptomatic presentations greatly undermine the effectiveness of vaccines, drastically stunting the overall process. Availability of specific testing lab and drugs are also sometimes challenging. In addition, basic medical interventions such as the administration of antibiotics in fully vaccinated individuals have shown to be effective in preventing transmission in 25% of symptomatic cases.^3^ However, despite all this, countries such as Nigeria continue to record the incessant incidence of diphtheria in the country. We speculate that different factors such as reduced awareness and campaigns on diphtheria, including low socioeconomic status, inadequate income, conflicts and war, inaccessibility to vaccination, including ineffective monitoring of the immunization schedule, as well as the emergence of the corona virus disease 2019 (COVID‐19) pandemic, could have led to the recent sporadic outbreaks of diphtheria in Nigeria. Owning to these problems, this present study therefore aimed to proffer a possible multifaceted approach to tackle outbreaks of diphtheria in Nigeria and improve immunization rates against the disease among the Nigerian population.

## 2 RECENT DIPHTHERIA OUTBREAKS AND VACCINATION EFFORTS IN NIGERIA, 2023

In 2011, Nigeria faced one of the most devastating outbreaks of diphtheria in Nigeria, especially Borno state (one of the northeastern states amid Boko‐Haram insurgencies), mainly affecting children between 0 and 4 years of age and with a fatality rate reaching 21.4%.^2^ Delayed clinical presentation, low immunization rates and lack of antibiotic treatment were identified as the leading causes of these outbreaks of diphtheria.^4^ Eleven years later (2011–2022), in December 2022, the Nigeria Centre for Disease Control and Prevention (NCDC) reported suspected diphtheria outbreaks in Kano and Lagos States. The NCDC made it known to the general public that from May 14, 2022 to April 9, 2023, 1439 suspected cases of diphtheria occurred, of which 557 (39%) were confirmed, including 73 deaths among the confirmed cases given a case fatality ratio of 13%.

**Figure 1:**
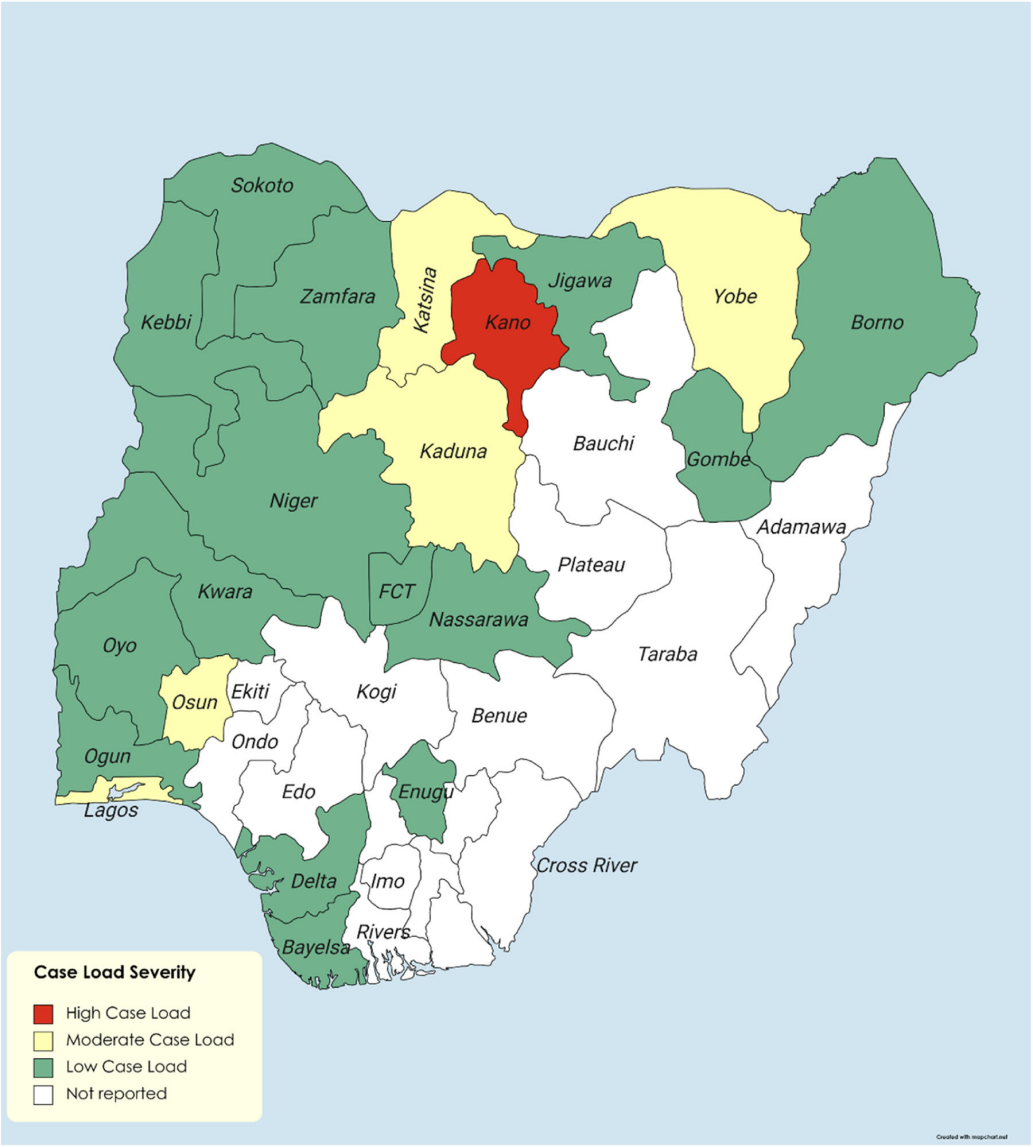
Case‐load severity of the recent diphtheria outbreak in Nigerian states.

Sadly, from June 30 to August 31, 2023, a total of 5898 suspected cases of diphtheria were reported from 59 local government areas in 11 new states in Nigeria, of which a total of 4717 confirmed cases of the disease was reported; 3466 (73.5%) were aged 1–14 years; of these, 699 were aged 0–4 years, 1505 were aged 5–9 years, and 1262 were aged 10–14 years with a case fatality of 6.1%. More than half of the cases (2656; 56.3%) were females. Unfortunately, only 1074 (22.8%) of the confirmed cases of diphtheria were fully vaccinated against diphtheria, whereas 299 (6.3%) were partially vaccinated.^5^, ^6^ See Figure 1 below.

Recently, Oduoye et al.^7^ found that a confirmed case of diphtheria was admitted at the emergency pediatric unit of Ahmadu Bello University Teaching Hospital, Kaduna state, on January 22, 2023, with another case admitted through contact tracing. Both victims of the disease were said to be living together in the same house.^7^ Not only in Nigeria, other African Union Member states such as Algeria, Guinea, and Niger, the cases are also increasing. As of October 9, 2023, 14,578 cases had been detected with a CFR of 4.1%.^8^ Poor vaccination rate and lack of herd immunity are a major cause of this outbreak. In addition, incidence rate in female is higher, so proper community screening is important. In resource restricted setup of African countries, it is challenging to combat the situation. Antibiotics such as Azithromycin and Amoxicillin play the key role in the treatment.

Due to multiple ongoing public health emergencies such as Lassa fever, cholera, and monkeypox in Nigeria, this outbreak of diphtheria further burdens the overstretched sources in Nigeria.^1^ The World Health Organization (WHO) has recently declared the frequent outbreaks of diphtheria in Nigeria as a public health concern.^2^, ^5^ Although vaccination efforts have been successful in Nigeria, unfortunately, it was estimated that almost seven million people remain unvaccinated and are at risk for infection in the country especially people living in the Northern part of the country.^7^ Of course, cases of diphtheria incidence are under‐reported in Nigeria.^7^ Although data might suggest a general decrease in the number of reported cases in Nigeria,^9^ 11 years’ worth of data remains missing in the WHO/United Nations Children's Fund (UNICEF) joint reporting process raising serious concerns over the management techniques. What's more is that of the cases reported in scientific literature, nearly all of them unanimously reflect the country's poor monitoring and reporting system.^9^, ^10^

It is obvious that the recent outbreaks of diphtheria in Nigeria despite being a vaccine‐preventable disease (VPD), reflect a major failure in the comprehensive Nigerian Program on Immunization (NPI) schedule in the country^7^ and, of course, a really big mess to the Nigerian health system and certainly a menace to ponder on. It is not surprising given that the COVID‐19 pandemic has disrupted health programs worldwide^11^ and could be a threat to the Nigerian health system.^7^

The shocking news is the large number of cases of diphtheria that simply went under the radar for either not being related to the disease (7.1%), not being classified properly (15.3%), or simply due to an uncertain status (33.2%).^2^, ^5^ Of these, it is important to note that children and adolescents accounted for the vast majority (91.9%). With only 22.8% of those afflicted having received full vaccination with a diphtheria toxin‐containing vaccine.^5^

### 2.1 Addressing diphtheria outbreak in Nigeria through multifaceted strategies and future directions

The recent outbreak of diphtheria is an alarming reminder of why diseases that are otherwise rare in the developed world continue to be an emerging cause for concern in a country like Nigeria. The country's poor general immunization status and narrow healthcare coverage with only 43% of citizens at access^12^ puts their young population in the crosshairs of such recurrent outbreaks. It is important to make a vaccination drive in Nigeria. Vaccination should be done from every community level. Vaccination centers should be open in every 500 meters. Rural hospitals should do monthly meetings on vaccinations. Health workers should go to every house and they should list the unvaccinated people. Further, those people should be vaccinated as early as possible. Next important part is vaccine storage. Cold chain method must be incorporate strongly. Public health personals should train the health care workers about cold chain mechanism and strict surveillance on the procedure in important. Every death due to diphtheria should be reviewed in the death review meeting and policies should be changed if needed. Moreover, the absence of DAT, Boko‐Haram insurgencies, banditries, kidnapping and other social vices in Nigeria further aggravates the gravity of the situation by heralding a terrible prognosis for a possibly fatal disease. Police should be very active on these incidences. DAT and other antibiotics should be stored properly in the higher incidence areas. Screening has a major role. Each and every person should be screened. Community and block medical officers should conduct an constant surveillance on screening. Major labs should be created for the tests. Females are very much neglected in the societies, so screening in female gender is crucial. It should also be noted that Nigeria, with its strategic location in the African region and a booming population of 213.4 million,^13^ puts its neighboring countries such as Niger Republic, Benin Republic, Cameroon, and so on, at as much risk of infectious outbreaks as well with reports of re‐infections being well‐documented in the past.^14^ Traveling Standard Operating Protocol should be created. Screening of travelers must be mandatory. Unvaccinated travelers should be restricted from boarding. Novel treatment such as exosome‐based treatments should be incorporated.^15^

If Nigeria is to emerge beyond these frequent epidemics of diphtheria, the Nigerian government, health authorities, policy‐makers, Nigerian citizens, as well as the international community must work on tackling this issue on multiple fronts simultaneously. Proper community‐based awareness is important. Media and social medias are currently the best platforms for mass awareness. Government can start some incentives on vaccinations. These indicate that multifaceted efforts to eradicate diphtheria in Nigeria can be classified as national efforts and international efforts. National efforts would entail the improvement of the NPI by the Nigerian government such as the provision of free DAT vaccines nationwide, training more vaccine experts on how to administer vaccines without a glitch, working with religious and traditional institutions, and relevant nonprofit organizations including students unions in Nigeria, as well as amnesty and peace organizations in the world, in putting an end to the political instabilities, insecurities, as well as the Boko‐Haram insurgencies in the country, to combat against diphtheria in the country. Also, the Nigerian government and nongovernment organizations in the country must work together towards propagating a wave of knowledge and awareness amongst the Nigerian population to overcome the shroud of vaccine hesitancy, be it in the form of media awareness or on‐site public education. Moreover, the Nigerian health authorities should allot sufficient monetary to improving vaccine delivery and coverage, drug availability in both rural and urban centers in the country, as well as provision of general infrastructure, social amenities (good source of water, good roads, constant power supplies, constant internet connection, and so on), and staffing of health care centers in both private and public hospitals in Nigeria.

For the Nigerian citizens, we advocate that Nigerian citizens should cooperate with the policy laid down on diphtheria control and prevention by the Nigerian government. Nigerian parents and guardians should take their children for immunization against childhood diseases like tuberculosis, measles, meningitis including diphtheria, and they should comply with the NPI schedule. These parents and guardians should report any diphtheria‐related symptoms such as fever, skin rashes, neck‐swelling, limb‐paralysis, and so on, to the nearest health facility for early diagnosis, treatment, and good prognosis. Oduoye et al.^7^ recommended that mothers and caregivers in Nigeria should be encouraged and educated by the Community Health Extension Workers, the physicians (especially the pediatricians), as well as the nurses in Nigeria about the importance of the diphtheria–tetanus–pertussis vaccine for their infants after 6 weeks of life. This vaccine should be received till the 14th week of life. Furthermore, healthcare providers, especially physicians and school teachers in Nigeria should emphasize that all mothers and caregivers in Nigeria should bring proof of vaccination (vaccination cards) when bringing their children to the hospitals and schools, respectively, during immunization exercise.^7^ Oduoye et al.^7^ believed that the approach would help to monitor the vaccination rate against diphtheria among infants in Nigeria.

Moving forward, part of the national efforts to unmask diphtheria in Nigeria also include the Nigerian health authorities through the physicians, nurses, and other paramedics to step up their awareness campaigns (through social media platforms, webinars, radio/television programs, and so on) about the importance of immunization against diphtheria and other VPDs among the Nigerian citizens including the religious and traditional leaders, market women leaders, student organizations and social groups, and so on in the country. This would improve their knowledge, attitude, and vaccination practice against infectious diseases including diphtheria. We think that there is a need to maintain high vaccination coverage against diphtheria and encourage booster doses that can aid in addressing DAT hesitancy and improving immunization rates among the Nigerian population.

Furthermore, collaboration between different sectors is crucial for an effective response to the diphtheria outbreak. Public and private bodies in Nigeria must foster partnerships in supporting vaccination campaigns, providing resources, and improving healthcare infrastructure in Nigeria. Both public and private bodies should encourage community leaders, religious institutions, and local influencers in Nigeria about the DAT vaccine. We believe that this public and private partnership would help to disseminate accurate information, address misconceptions, and promote the acceptance of vaccination against diphtheria and other VPDs in Nigeria. Buttressing this matter further, we recommend that Nigerian policymakers establish a robust surveillance system for diphtheria in Nigeria. Through robust surveillance of diphtheria, there would be a strengthening of capacity for laboratory confirmation of diphtheria, expanding the network of sentinel sites for data collection, and implementing a centralized reporting system on the disease in Nigeria. We are optimistic that timely and accurate surveillance data about diphtheria would inform evidence‐based decision‐making, enable early detection of outbreaks, and facilitate effective allocation of resources.

At the international level, the Nigerian Ministry of Health should collaborate with the WHO, UNICEF, United Nations Educational, Scientific and Cultural Organization, and United Nations Population Fund in establishing more training programs on immunization, emergency response, children's health, sexual and reproductive health, and so on among Nigerian healthcare workers to improve their knowledge, attitude, and perception in detecting and managing diphtheria cases including novel research and funding opportunities against diphtheria in Nigeria. This collaboration should also involve the improvement of all Nigerian laboratory capabilities for accurate diagnosis and treatment, as well as educating patients, including women and children on the proper use of antibiotics and antitoxin, while treating diphtheria patients. Furthermore, the Nigerian Ministry of Health should collaborate with the Nigerian Armed Forces, the Nigerian Police Force, the Nigerian Navy, as well as Vigilante local groups in providing adequate security for medical personnel in the country, especially the states that are mostly affected by social vices, political instability, Boko‐Haram insurgencies, and so on. This would prevent them from being attacked while carrying out vaccination exercises and would also boost their willingness and confidence level to help victims of diphtheria in those states as well as increase wide vaccination coverage.

## 3 CONCLUSION

In conclusion, we have strong opinions that our recommendations will go a long way towards improving medical preparedness against diphtheria in Nigeria. Health reporting and monitoring systems must also be revamped and made more effective to facilitate prompt notification to both national and international health bodies. The Nigerian government must facilitate the coverage of vaccines, making sure healthcare personnel are provided with adequate safety and security when accessing non‐state armed areas. Finally, strict public measures must be instituted to condone and limit the respiratory transmission of this disease. To this end, the Nigerian government and the general public must work together to achieve a diphtheria‐free Nigeria.

## AUTHOR CONTRIBUTIONS

**Malik O. Oduoye**: Conceptualization, data curation, writing—original draft, writing—review and editing. **Mohammed Dheyaa Marsool Marsool**: Writing—original draft, writing—review and editing. **Muhammad Usman Haider**: Writing—original draft, writing—review and editing. **Dattatreya Mukherjee**: Writing—original draft, writing—review and editing. **Aimen Waqar Khan**: Writing—original draft, writing—review and editing. **Temidayo O. Faloye**: Writing—original draft, writing—review and editing. **Minal Jehan**: Writing—original draft, writing—review and editing. **Abdullahi A. Adegoke**: Writing—original draft, writing—review and editing. **Karim A. Karim**: Writing—original draft, writing—review and editing. **Tirth Dave**: Project administration, supervision, writing—original draft, writing—review and editing.

## CONFLICT OF INTEREST STATEMENT

The authors declare no conflict of interest.

## TRANSPARENCY STATEMENT

The lead author Tirth Dave affirms that this manuscript is an honest, accurate, and transparent account of the study being reported; that no important aspects of the study have been omitted; and that any discrepancies from the study as planned (and, if relevant, registered) have been explained.

## Data Availability

The data used in this study are publicly available from the National Library of Medicine https://www.ncbi.nlm.nih.gov/. All relevant data are included in the manuscript. Additional data related to this study may be requested from the corresponding author upon reasonable request.
